# Supplementary material for: Association between serum β2-microglobulin and mortality in Japanese peritoneal dialysis patients: A cohort study
Source: PLoS One. 2022 Apr 14;17(4):e0266882. doi: 10.1371/journal.pone.0266882 (PMC9009671; doi:10.1371/journal.pone.0266882)
Supplement: S1 Table — (DOCX) [file pone.0266882.s001.docx]

**S1 Table. Comparisons of baseline characteristics between patients with and without serum B2M data.**

| Variable | Total | With B2M data | Without B2M data | P |
| --- | --- | --- | --- | --- |
| Number (%) | 6883 | 3011 (43.7%) | 3872 (56.3%) |  |
| Age (years) | 62±14 | 63±13 | 62±14 | 0.16 |
| Male (%) | 4194 (60.9%) | 1848 (61.4%) | 2346 (60.6%) | 0.52 |
| Dialysis duration (months) | 30 (14-55) | 31 (15-54) | 30 (14-56) | 0.87 |
| Height (cm) | 160±9 | 160±9 | 160±10 | 0.21 |
| BW (kg) | 59.8±12.5 | 60.1±12.4 | 59.3±12.5 | 0.07 |
| BMI (kg/m^2^) | 23.1±3.7 | 23.2±3.7 | 23.0±3.7 | 0.20 |
| Underlying disease |  |  |  | <0.01 |
| CGN (%) | 2869 (41.7%) | 1301 (43.2%) | 1568 (40.5%) |  |
| Diabetic nephropathy (%) | 1955 (28.4%) | 856 (28.4%) | 1099 (28.4%) |  |
| Nephrosclerosis (%) | 788 (11.4%) | 362 (12.0%) | 426 (11.0%) |  |
| PKD (%) | 160 (2.3%) | 76 (2.5%) | 84 (2.2%) |  |
| Others or unknown (%) | 1111 (16.1%) | 416 (13.8%) | 695 (17.9%) |  |
| Comorbidity |  |  |  |  |
| AMI | 305 (6.9%) | 182 (6.6%) | 123 (7.4%) | 0.34 |
| Cerebral hemorrhage | 146 (3.3%) | 88 (3.2%) | 58 (3.5%) | 0.60 |
| Cerebral infarction | 501 (11.3%) | 290 (10.5%) | 211 (12.6%) | 0.04 |
| Quadruple amputation | 47 (1.1%) | 25 (0.9%) | 22 (1.3%) | 0.18 |
| Laboratory data |  |  |  |  |
| B2M (mg/L) | N.A. | 24.6 (18.4-33.2) | N.A. | N.A. |
| BUN (mg/dL) | 54±15 | 54±15 | 53±16 | 0.01 |
| Cr (mg/dL) | 9.5±3.3 | 9.6±3.3 | 9.4±3.4 | <0.01 |
| Alb (g/L) | 3.3±0.5 | 3.4±0.5 | 3.3±0.6 | <0.01 |
| CRP (mg/dL) | 0.1 (0-0.5) | 0.1 (0-0.5) | 0.1 (0-0.5) | 0.02 |
| Hb (g/dL) | 10.4±1.5 | 10.4±1.5 | 10.2±1.5 | <0.01 |
| PD related parameters |  |  |  |  |
| D/P Cr | 0.66±0.13 | 0.66±0.13 | 0.66±0.14 | 0.99 |
| Use of icodextrin | 865 (22.3%) | 533 (21.8%) | 332 (23.0%) | 0.40 |
| UV (mL/day) | 600 (200-1000) | 650 (200-1030) | 600 (200-1000) | 0.09 |
| Renal Kt/V | 0.5 (0.1-0.9) | 0.5 (0.1-0.8) | 0.5 (0.1-0.9) | 0.11 |
| PD Kt/V | 1.4 (1.0-1.7) | 1.3 (1.0-1.7) | 1.4 (1.0-1.7) | <0.01 |
| History of PD peritonitis | 637 (17.8%) | 387 (17.3%) | 250 (18.6%) | 0.34 |
|  |  |  |  |  |
| Date is shown as means ± SD or medians and interquartile ranges (IQR). | | |  |  |
| Abbreviations: B2M, β2 microglobulin; BW, body weight; BMI, body mass index; CGN, chronic glomerulonephritis; PKD, polycystic kidney disease; AMI, acute myocardial infarction; BUN, blood urea nitrogen; Cr, creatinine; Alb, albumin; CRP, C reactive protein; Hb, hemoglobin; PD, peritoneal dialysis; D/P Cr, dialysate-to-plasma ratio of creatinine; UV, urinary volume. | | | | |
