# Supplementary material for: Association between serum β2-microglobulin and mortality in Japanese peritoneal dialysis patients: A cohort study
Source: PLoS One. 2022 Apr 14;17(4):e0266882. doi: 10.1371/journal.pone.0266882 (PMC9009671; doi:10.1371/journal.pone.0266882)
Supplement: S2 Table — (DOCX) [file pone.0266882.s002.docx]

**S2 Table. HR and 95% CI of all-cause mortality and SHR and 95% CI of CV mortality, with analysis using serum B2M as a categorical variable.**

|  |  |  |  |  |  |
| --- | --- | --- | --- | --- | --- |
|  | HR and 95% CI of all-cause mortality | |  | SHR and 95% CI of CV mortality | |
|  | Unadjusted | Adjusted |  | Unadjusted | Adjusted |
| Male gender | 1.17 (1.04 to 1.31) | 1.46 (1.28 to 1.66) |  | 1.21 (0.99 to 1.47) | 1.22 (0.98 to 1.52) |
| Age [yr] | 1.08 (1.07 to 1.08) | 1.07 (1.06 to 1.07) |  | 1.04 (1.04 to 1.05) | 1.04 (1.03 to 1.05) |
| Ln Dialysis duration [months] | 1.03 (0.97 to 1.10) | 1.17 (1.08 to 1.26) |  | 0.96 (0.87 to 1.05) | 1.09 (0.96 to 1.24) |
| Diabetes | 1.66 (1.48 to 1.87) | 1.49 (1.32 to 1.69) |  | 1.99 (1.65 to 2.41) | 1.79 (1.46 to 2.21) |
| BMI [kg/m^2^] | 0.98 (0.96 to 0.999) | 1.00 (0.98 to 1.02) |  | 1.01 (0.98 to 1.04) | 1.01 (0.98 to 1.04) |
| BUN [mg/dL] | 0.991 (0.987 to 0.994) | 1.006 (1.002 to 1.010) |  | 0.993 (0.987 to 0.999) | 0.999 (0.992 to 1.006) |
| Cr [mg/dL] | 0.88 (0.86 to 0.89) | 0.89 (0.86 to 0.91) |  | 0.92 (0.89 to 0.95) | 0.97 (0.92 to 1.02) |
| B2M [mg/L] |  |  |  |  |  |
| < 18.5 | 1.08 (0.92 to 1.27) | 0.98 (0.83 to 1.17) |  | 0.92 (0.71 to 1.20) | 0.90 (0.68 to 1.20) |
| 18.5 to < 24.7 | Ref. | Ref. |  | Ref. | Ref. |
| 24.7 to < 33.3 | 1.12 (0.95 to 1.31) | 1.33 (1.12 to 1.58) |  | 1.07 (0.83 to 1.38) | 1.17 (0.89 to 1.56) |
| ≥ 33.3 | 1.15 (0.98 to 1.34) | 1.71 (1.40 to 2.08) |  | 0.90 (0.69 to 1.18) | 1.19 (0.84 to 1.69) |
| Alb [g/dL] | 0.35 (0.31 to 0.39) | 0.61 (0.53 to 0.70) |  | 0.53 (0.44 to 0.63) | 0.81 (0.64 to 1.01) |
| Ln CRP [mg/dL] | 1.24 (1.20 to 1.28) | 1.11 (1.07 to 1.14) |  | 1.17 (1.11 to 1.24) | 1.08 (1.02 to 1.14) |
| Hb [g/dL] | 0.93 (0.90 to 0.97) | 0.97 (0.93 to 1.01) |  | 0.93 (0.87 to 0.99) | 0.96 (0.89 to 1.03) |
| History of AMI | 2.51 (2.08 to 3.03) | 1.31 (1.08 to 1.60) |  | 2.13 (1.57 to 2.89) | 1.35 (0.96 to 1.90) |
| History of cerebral bleeding | 1.82 (1.38 to 2.40) | 1.38 (1.03 to 1.85) |  | 1.68 (1.06 to 2.65) | 1.17 (0.71 to 1.95) |
| History of cerebral infarction | 2.53 (2.17 to 2.96) | 1.27 (1.07 to 1.49) |  | 1.70 (1.29 to 2.24) | 1.04 (0.78 to 1.39) |
| D/P Cr ratio | 2.53 (1.35 to 4.75) | 0.88 (0.46 to 1.68) |  | 1.63 (0.53 to 4.98) | 0.70 (0.27 to 1.82) |
| Use of icodextrin | 1.13 (0.97 to 1.32) | 1.03 (0.88 to 1.21) |  | 1.11 (0.86 to 1.43) | 1.03 (0.77 to 1.37) |
| Ln UV [mL/day] | 0.98 (0.96 to 1.01) | 0.99 (0.95 to 1.02) |  | 1.05 (0.99 to 1.11) | 1.06 (0.99 to 1.12) |
| History of PD peritonitis | 1.29 (1.09 to 1.52) | 0.94 (0.80 to 1.11) |  | 1.13 (0.85 to 1.51) | 0.95 (0.71 to 1.26) |
|  |  |  |  |  |  |
| Abbreviations: HR, hazard ratio; CI, confidence interval; SHR, sub-hazard ratio; CV, cardiovascular; B2M, β2 microglobulin; BMI, body mass index; BUN, blood urea nitrogen; Cr, creatinine; Alb, albumin; CRP, C-reactive protein; Hb, hemoglobin; AMI, acute myocardial infarction; D/P Cr, dialysate-to-plasma ratio of creatinine; UV, urinary volume; PD, peritoneal dialysis. | | | | | |
